# Lower Choline-Containing Metabolites/Creatine (Cr) Rise and Failure to Sustain NAA/Cr Levels in the Dorsolateral Prefrontal Cortex Are Associated with Depressive Episode Recurrence under Maintenance Therapy: A Proton Magnetic Resonance Spectroscopy Retrospective Cohort Study

**DOI:** 10.3389/fpsyt.2017.00277

**Published:** 2017-12-13

**Authors:** Neven Henigsberg, Helena Šarac, Marko Radoš, Milan Radoš, David Ozretić, Tamara Foro, Viktorija Erdeljić Turk, Pero Hrabač, Maja Bajs Janović, Benedict Rak, Petra Kalember

**Affiliations:** ^1^School of Medicine, University of Zagreb, Zagreb, Croatia; ^2^University Hospital Centre Zagreb, Zagreb, Croatia; ^3^Polyclinic Neuron, Zagreb, Croatia; ^4^University Hospital “Sveti Duh”, Zagreb, Croatia

**Keywords:** depression, recurrence, maintenance therapy, antidepressants, magnetic resonance spectroscopy

## Abstract

**Background:**

The aim of this study was to evaluate the relationship between changes in proton magnetic resonance spectroscopy (1H-MRS) parameters at the start of the index episode recovery phase and at recurrence in patients with recurrent depression who were treated with prolonged maintenance therapy.

**Methods:**

1H-MRS parameters were analyzed in 48 patients with recurrent depression who required maintenance therapy with antidepressant medication prescribed by a psychiatrist and who continued with the same antidepressant during the maintenance phase, either to recurrence of depression, completion of the 10-year observation period, or the start of the withdrawal phase (tapering-off antidepressant). N-acetylaspartate (NAA), choline-containing metabolites (Cho), creatine (Cr), and glutamine/glutamate were measured at the start of the recovery phase and 6 months later.

**Results:**

Recurrent depressive episodes occurred in 20 patients. These individuals had a smaller increase in Cho/Cr after the beginning of the recovery phase compared to the non-recurrent patient group and also exhibited a decreased NAA/Cr ratio.

**Conclusion:**

Sustainable NAA and increased Cho levels at the onset of the recovery phase of the index episode are early markers of antidepressant effectiveness associated with a lower risk of major depressive disorder recurrence. The NAA and Cho changes in the non-recurrent group may be attributable to increased brain resilience, contrary to the transient temporal effect observed in subjects who experienced a depressive episode.

## Introduction

Approximately 35% of depressed patients experience a recurrent episode. The longitudinal course includes an active phase, which is followed by remission and recovery phases. A patient is considered to be in remission after 3 consecutive weeks of maintaining minimal symptom status. They are in recovery, if a relapse does not occur for at least 4 months followed by the onset of remission. Recovery, once achieved, is lost if followed by a recurrence (1). In a 10-year follow-up study, the risk of the recurrence of major depressive disorder (MDD) progressively increased with each successive episode. The risk fell as time without recurrence elapsed, whereas the within-subject time to recurrence varies considerably from one recurrence to the other (2). Other identified risk factors for recurrence include family history of recurrent depression, previous history of dysthymic disorder, early onset, and residual depressive symptoms (3). After three episodes, the risk of recurrence approaches 100% in the absence of prophylactic treatment; patients who have had three or more prior major depressive episodes should proceed to the maintenance phase of therapy after completing the continuation phase (3). Antidepressants should be prescribed at the full therapeutic dose who achieved symptom remission during the acute and continuation phases. Patients should be monitored at regular intervals using standardized measurement aids to facilitate early detection of recurrent symptoms.

In larger studies, the average episode duration ranges from 16 to 24.3 weeks (4, 5). With regard to pharmacotherapies, a meta-analysis of 102 studies found no overall difference in efficacy between selective serotonin reuptake inhibitors (SSRIs) and tricyclic antidepressants (TCAs) (4).

Proton magnetic resonance spectroscopy (1H-MRS) evaluates brain metabolites *in vivo* and could improve our understanding of molecular and submolecular changes in the brain. The 1H-MRS technique is based on the fact that different molecules possess unique magnetic resonance (MR) spectra, and their quantities can be measured against standard metabolite curves. Among the spectroscopic techniques, proton spectroscopy is the most widely used due to its high sensitivity, distinguishable spectra, high signal-to-noise ratio, and ease of use as this technique can be performed using standard radiofrequency coils (5). Neurochemicals and complexes identified by 1H-MRS, include N-acetylaspartate (NAA), creatine (Cr), choline-containing metabolites (Cho), glutamine/glutamate (Glx), myoinositol, glycine, glucose, lipids, lactate, alanine, acetate, succinate, and taurine.

Since NAA is found in neurons, but not in glial cells or blood, it is considered a putative marker of neuronal integrity (6) and functionality (7, 8). Several studies have shown altered NAA levels following antidepressant treatment. Increased NAA/Cr ratios were reported in the prefrontal cortex (PFC) and frontal white matter after SSRI therapy (9), as well as in the hippocampus (10). Another group reported higher NAA in the left medial frontal cortex of depressed patients after treatment with SSRIs and serotonin–norepinephrine reuptake inhibitors (11). NAA changes were observed even after just 7 days of treatment (9). A voxel-based morphometric study (12) reported reduced gray matter volume in the left dorsolateral prefrontal cortex (DLPFC) of non-remitting MDD patients, and another group concluded that a longer history of depression correlates with lower NAA values (13). Collectively, these results support the hypothesis that illness chronicity leads to neurodegenerative processes in the DLPFC.

Glycerophosphocholine (GPC) and phosphocholine (PC) represent over 50% of cytosolic Cho-containing compounds in the living brain (14, 15). As PC is a precursor of membrane phospholipids and GPC is a breakdown product (16), Cho is considered a potential marker of membrane phospholipid metabolism and membrane turnover. Increased Cho/Cr ratios in the bilateral ventral prefrontal white matter of MDD patients was documented after 12 weeks of SSRI treatment (17). We previously reported a higher Cho/Cr ratio in the absence of any change in NAA/Cr in the DLPFC in early-to-intermediate responders after 3–6 weeks of SSRI treatment for recurrent depression and comorbid posttraumatic stress disorder (18). The increase of Cho in therapy responders does not seem to be medication specific, as the increase is also observed in PFC after sleep restriction (19) and repetitive transcranial magnetic stimulation (20).

Research of glutamine, glutamate, and gamma-aminobutyric acid (GABA) metabolites, usually combined in a Glx peak, is based on the observation of GABAergic dysfunction in depressed subjects who have low plasma and cerebrospinal fluid GABA concentrations (21). A meta-analysis reported a reduction in absolute Glx values in the PFC of patients with depression, correlating with disease severity (22). GABA levels in MDD patients are also lower, but normalize following antidepressant treatment (23), so remitted MDD patients do not have significantly different values from controls (24).

The DLPFC is a particular region of interest (ROI) since neuroimaging, lesion analysis, postmortem, histopathological, and genomic studies have consistently indicated abnormalities in this brain area of depressed patients, whereas hyperactivity has been reported in the DLPFC during the recovery phase in response to medication (25). A recent review emphasized the need for further research of the DLPFC and other frontal regions over a longer period, to establish the functional relevance of Cho metabolites in the treatment of MDD (26).

The aim of this study was to evaluate the relationship between changes in 1H-MRS parameters at the start of the index episode recovery phase, and during recurrence in patients suffering from recurrent depression, who were prescribed maintenance therapy. Our approach is unique in that we correlated changes in 1H-MRS parameters coincident with the recovery period with clinical outcomes. Based on the literature on changes in 1H-MRS parameters over the course of treatment, we hypothesized that patients who maintained stable condition (i.e., begun tapering-off medication without a recurrence) would exhibit differences in Cho levels and possibly also in NAA and Glx ratios.

## Materials and Methods

This was a retrospective cohort study. Among patients who previously participated in the past research into predictors of therapeutic response, we identified 48 patients who had MRS scans of the DLPFC at the start of the recovery period and 6 months later. The primary goal of this study was to describe MRS metabolite changes between the time of the recovery and after 6 months, but the results were inconclusive and considered inappropriate for publication. The MDD patients included in this study had no history of comorbid psychiatric disorders. Diagnoses of depression and recurrent depression were based on International Classification of Diseases (ICD)-10 diagnostic criteria and were confirmed by Mini International Neuropsychiatric Interview (MINI) 5.0 (27). A comorbid diagnosis of somatic illness was the exclusion criterion. This study was approved by the institutional ethics committee, and this study protocol was formulated in accordance with the Declaration of Helsinki. Prior to their inclusion, patients had already consented to participate in long-term evaluations that would include psychiatric interviews and assessments. Both were performed at least every 6 months (and occasionally even more frequently, as patients opted for regular visits due to local formal procedures requiring a patient to attend a psychiatric evaluation to obtain prescriptions within the obligatory health insurance scheme). Throughout the study, all patients were treated on an outpatient basis. Monotherapy with antidepressants and a stable dose of medication from the benzodiazepine class, if used, was set as an inclusion criterion when recovered patients were selected for the MRS study. Patients were treated only with pharmacotherapy during the research period. Psychotherapy and any other therapy methods were not used.

The choice of antidepressant medication was left to the practitioner treating the patient, and no conditions were set either with regard to the need for changing the antidepressant or the therapy duration. Participants were prescribed antidepressants according to the Summary of Product Characteristics of the individual medication. Baseline evaluation was performed at the first contact with the patient in relation to the current episode, after the patient consented to participate in this study. Definitions of remission and recovery were based on the standard criterion of a Montgomery–Asberg Depression Scale (MADRS) score ≤10. MRS scanning in the recovery phase was performed the same day as the clinical evaluation or 2 days after at the latest and at least 4 months after the onset of remission, when the MADRS score was ≤10.

Patients were followed up either until the beginning of the tapering phase of the antidepressant medication or to the emergence of recurrent depression symptoms. In addition to regular psychiatric assessments, regular 6-month follow-ups included MADRS scale evaluation. We use the term “withdrawal” in this study to indicate the first time point in the maintenance period when the dose of antidepressant medication was changed (tapered) with the intent to completely discontinue medication treatment. The withdrawal phase starts at this time point, and the future course of illness is beyond the scope of this study.

**Figure 1:**
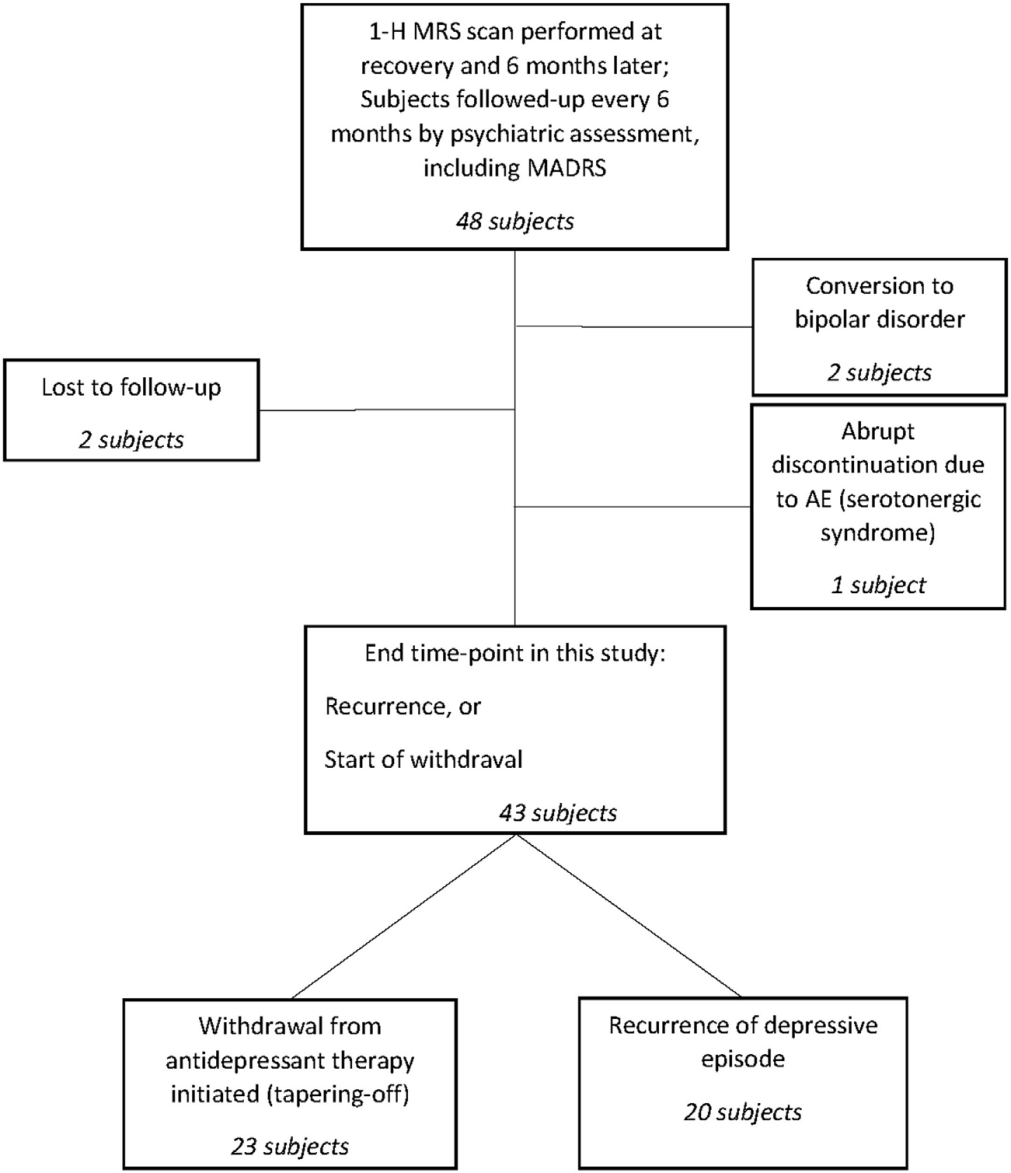
Study schematic.

The schematic study is displayed in Figure 1.

### 1H-MRS Analyses

Proton magnetic resonance spectroscopy data acquisition was performed using a 2.0 T system (Gyrex 2T-Prestige, GEMS/Elscint, Haifa, Israel) with a quadrate head coil. The subject was in a supine position, with the intersection of the frontal bone and two nasal bones (nasion) serving as a landmark. Foam pads were used to minimize head motion. Voxels were placed for spectroscopy, and all data analyses were performed by a trained radiologist blinded to the subjects’ diagnoses. Standard T1 and T2 sequences completed in the coronal, sagittal, and axial planes and covering the entire brain were acquired for each subject to assess the effect of spectral localization and exclude possible structural brain damage. The voxels were repositioned in the ROI of the left DLPFC. The spectroscopic volume of interest, 15 mm × 15 mm × 15 mm, was selected in the left DLPFC region to minimize the amount of cerebrospinal fluid contained in the volume of interest. 1H-MRS was performed using a point-resolved spectroscopy sequence (1,500/35 [repetition time/echo time]), with 100 averages. Each spectrum was re-evaluated for peak NAA (at 2.02 ppm), Cho (at 3.2 ppm), Glx (at 2.2–2.4 ppm), and Cr (at 3.03 ppm). The absolute NAA, Cho, and Glx values and their ratios to Cr were used for the analyses. Analyses of the spectral dataset were performed using the software package program supplied by the manufacturer of the MR system (Gyrex 2T-Prestige, GEMS/Elscint).

### Statistical Analysis

Statistical analysis was performed with the Statistica software package, version 13.2 (Dell Inc., Austin, TX, USA). The level of statistical significance was set to 0.05 for all analyses. The hypothesis in relation to power analysis was that groups would differ in Cho/Cr changes between the start of recovery period and the 6-month follow-up. With estimated between group differences in Cho/Cr ratio changes of 0.05 and a SD of 0.1, where α = 0.05, a sample size of 43 with 1.15 sampling ratio would achieve power (1 − β) of 0.67. Before performing any analyses, the normality of distribution of continuous variables was assessed with both Kolmogorov–Smirnov and Shapiro–Wilk tests. Differences between the two subject groups were first analyzed by means of Student’s *t*-tests or Mann–Whitney *U* tests. Differences in categorical variables were assessed with χ*^2^* tests.

Descriptive statistics are displayed for 43 subjects who either experienced a recurrent episode on maintenance therapy (20 subjects) or entered the withdrawal phase. Five remaining patients were excluded from descriptive analyses, since the future direction of change of their remission is unknown (two patients were lost to further follow-up) or were assessed at the 6-month follow-up after the recovery phase and later received in-patient treatment (two bipolar patients and one patient with serotonergic syndrome). However, final observations from those five patients are useful for survival estimations and their observations were entered into the analysis as censored cases.

All cases except those with documented episodes that occurred after the index episodes are censored. The most frequent reason was the initiation of maintenance therapy withdrawal. In that group, it would not be prudent to record the possible later onset of the episode as the one that occurred under maintenance therapy, since the maintenance antidepressant dose was already decreased, if not completely withdrawn, prior to the episode.

We employed Cox proportional hazard (CPH) analysis using a forward stepwise likelihood ratio model to assess neurochemical brain changes as prognostic risk factors for depression recurrence. The prognostic factors to be entered into CPH analysis involved brain metabolites in the DLPFC, those based on current knowledge regarding the recurrence of depressive episodes under maintenance therapy, and the set of factors we assumed might modify metabolite levels. The initial set of variables includes post-recovery changes in NAA/Cr, Cho/Cr, and Glx/Cr metabolites as our primary variables of interest, as well as the time elapsed between remission and baseline, age at first episode onset, years of depression, the number of prior depressive episodes, MADRS score at baseline, improvement on MADRS score at recovery, and MADRS score change between recovery and 6-month follow-up. The baseline MADRS score was highly correlated with improvement in MADRS score at recovery (*r* = 0.96), so we only kept MADRS improvement at recovery in the model.

The model entered into the CPH analysis initially consisted of three MRS metabolites, three variables describing the course of a current episode (time to remission, MADRS improvement at recovery, and change in the MADRS score between recovery and 6-month follow-up), and three variables describing the past course of recurrent depressive disorder (age at the onset of the first episode, years of depression, and number of prior episodes).

After identifying the CPH model that contained parameters with significant contributions, we developed a model with dichotomized variables in which we analyzed the overall direction of change in neurometabolites (<0 vs. ≥0), but not the extent of their rise or fall. We considered that this dichotomized model would enable easier interpretation and allow for the identification of significant concomitant changes in brain metabolites in relation to prognostic risk.

## Results

Forty-eight subjects were included in our study and were evaluated by 1H-MRS scans of the DLPFC at the start of their recovery period and 6 months later. Among these, 43 were followed up at regular 6-month intervals either to the recurrence of another depressive episode or until entering the medication tapering-off period. The remaining five subjects lacked consistent data until those end points, as they were either lost to follow-up (two patients), converted to bipolar disorder (two patients), or abruptly stopped medication due to an adverse event (serotonergic syndrome in one patient).

In total, 30 female and 18 male patients were included. Eleven (55%) female patients experienced another depressive episode while on maintenance therapy, and 15 (65.2%) entered the tapering-off period. The group of patients for which the resolution of maintenance therapy is not known consists predominantly of female patients (four females out of five patients). At the beginning of an index episode, the average age of patients who would later experience recurrence was 43.6 years (SD = 11.6), while the mean age of patients who started a withdrawal and did not experience subsequent episode was 44.5 years (SD = 13.2). No gender (χ^2^ = 0.47; d.f. = 1; *p* = 0.49) or age differences (*t* = 0.23; d.f. = 41; *p* = 0.82) were observed between the two groups. The most distant end point to which any patient was monitored was 6.9 years, considerably below the 10-year targets.

Patients who have entered the withdrawal phase were in the study nearly twice as long as those who experienced a subsequent episode during the study period (3.0 years; SD = 1.7 vs. 1.6 years; SD = 0.9; *p* = 0.0017), which is expected in a study of this type where patients experiencing the event are moved to the uncensored group. Another expected difference was the imbalance in depression severity at the last observation when all uncensored subjects were found to have worsening symptoms. However, that visit immediately preceded the diagnosis of a recurrent episode, so both groups had similar symptom severity (MADRS scores of 5.3; SD = 1.1 vs. 5.4; SD = 1.3), as displayed in Table 1. The groups were comparable by all other analyzed descriptors of disorder course and the current episode.

**Table 1:** Descriptors of courses of disorder and the current episode.

	Withdrawal started *n* = 23		Recurrence of an episode *n* = 20		
Variable	Mean	SD	Mean	SD	*p*
**Years to event**					
Withdrawal	3.03	1.67			
Recurrence			1.63	0.88	0.0017
**Disorder course descriptors**					
Age at onset of disorder	28.21	7.75	27.59	8.98	0.8088
Years of depression	16.27	10.15	15.99	11.13	0.9328
No. of prior episodes	3.78	1.73	4.00	2.41	0.7332
**Current episode descriptors**					
Months to remission	5.93	1.40	5.81	1.65	0.7945
**MADRS score**					
At the beginning of an episode	25.79	4.13	25.90	4.07	0.9305
At the start of the recovery phase	5.40	1.33	5.61	1.07	0.5696
6 months after the start of the recovery phase	5.55	0.88	5.29	1.41	0.4575
At the visit prior to the last evaluation	5.34	1.10	5.44	1.27	0.7733
**At the last evaluation**					
Withdrawal	5.32	0.87			
Recurrence			20.30	1.44	<0.0001

*MADRS, Montgomery–Asberg Depression Scale*.

*Withdrawal-first time point over study when antidepressant medication was tapered with the intention to discontinue medication*.

**Figure 2:**
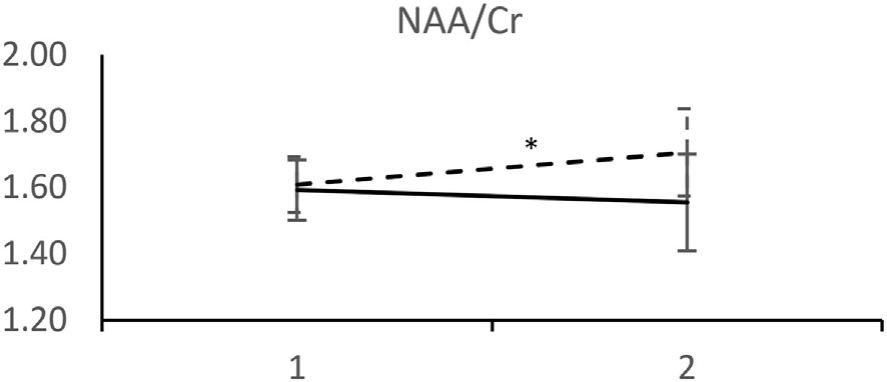
MRS measurements of NAA/Cr. (____) Followed-up until recurrence of depressive episode. (-----) Followed-up until start of antidepressant medication withdrawal. Horizontal axis: 1 – Start of the recovery phase; 2 – Start of the recovery phase + 6 months. Error bars denote 95%CI of means. *p < 0.005 *t*-test.

**Figure 3:**
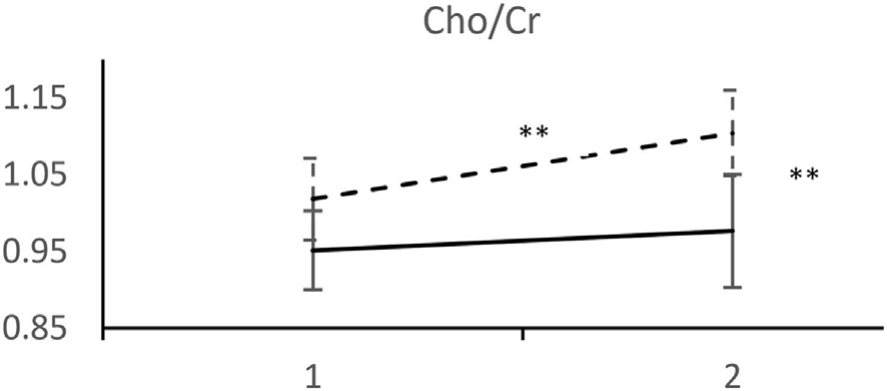
MRS measurements of Cho/Cr. (____) Followed-up until recurrence of depressive episode. (-----) Followed-up until start of antidepressant medication withdrawal. Horizontal axis: 1 – Start of the recovery phase; 2 – Start of the recovery phase + 6 months. Error bars denote 95%CI of means. **p < 0.001 *t*-test.

**Figure 4:**
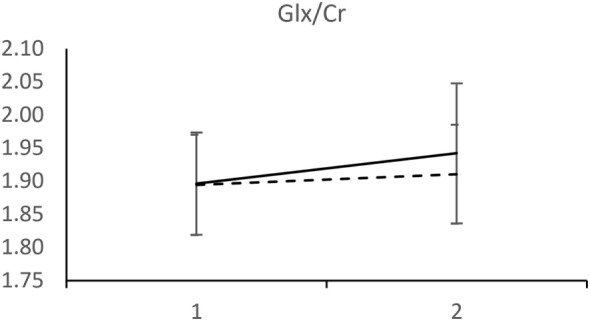
MRS measurements of Glx/Cr. (____) Followed-up until recurrence of depressive episode. (-----) Followed-up until start of antidepressant medication withdrawal. Horizontal axis: 1 – Start of the recovery phase; 2 – Start of the recovery phase + 6 months. Error bars denote 95%CI of means.

Analyzed 1H-MRS metabolite levels are characterized by two features in patients who experienced a recurrent depressive episode: a significant rise in NAA/Cr and even more pronounced rise in Cho/Cr (Figure 2). We observed an opposite direction of NAA/Cr change in patients with a recurrent episode (*t* = 2.11; d.f. = 41; *p* = 0.041) compared to those who remained in remission (Table 2). The change in Cho/Cr was more than threefold lower in patients who experienced a recurrent episode (*t* = 1.82; d.f. = 41; *p* = 0.076) (Figure 3). There was also nearly a threefold higher change in Glx/Cr in this group (*t* = –0.42; d.f. = 41; *p* = 0.676), but due to the high coefficient of variability, the result was not statistically significant (Figure 4).

**Table 2:** Changes in 1 H-MRS metabolite levels from the time of recovery to 6 months later.

	Withdrawal started *n* = 23		Recurrence of an episode *n* = 20		
Metabolite	Mean	SD	Mean	SD	p*
Cho/Cr	0.0860	0.0962	0.0250	0.1231	0.076
NAA/Cr	0.0972	0.2040	–0.0380	0.2155	0.041
Glx/Cr	0.0159	0.1958	0.0459	0.2690	0.676

*Two-sided t-test for independent samples.

*Cho, choline-containing metabolites; Cr, creatine; Glx, glutamate/glutamine; 1H-MRS, proton magnetic resonance imaging; NAA, N-acetylaspartate*.

*Withdrawal was the first time point over when antidepressant medication was tapered with the intention to discontinue medication*.

For every variable in the CPH analysis, we verified the proportional hazard assumption by constructing a product between the variable and a linear function of time, added an interaction term, and tested for its significance. This step was done to confirm that a time-varying effect was not present, as its existence would discredit the use of a CPH model and indicate that observed metabolite changes are correlates of an episode that has begun to neurochemically develop, but is not yet clinically manifested. None of the time-dependent covariates were significant. To assess the proportionality assumption, we also inspected a plot of the log–og[S(*t*)] functions and visually confirmed constant hazard ratios.

First, we evaluated the initial model which, in addition to neurometabolites of our interest, included variables from the literature seen as the basic indicators of the severity and longitudinal course of the current episode and as likely to increase the risk of recurrence. Parameter estimates of the initial model are presented in Table 3.

**Table 3:** Parameter estimates of the initial model.

Factor	Relative risk (95% confidence interval)	*p*
**1H-MRS metabolite changes 6 months after recovery**		
NAA/Cr	0.0639 (0.0062–0.6602)	0.0210
Cho/Cr	0.0077 (0.0001–0.5269)	0.0240
Glx/Cr	0.5544 (0.0744–4.1307)	0.5648

**Current episode descriptors**		
Months to remission	0.9031 (0.6171–1.3215)	0.5996
MADRS improvement at recovery	1.1034 (0.9664–1.2598)	0.1458
MADRS change 6 months after recovery	0.9378 (0.6637–1.3252)	0.7160

**Disorder course descriptors**		
Age at onset	0.993 (0.9299–1.0604)	0.8332
Years of depression	0.9644 (0.9036–1.0294)	0.2760
No. of prior episodes	1.116 (0.8407–1.4816)	0.4477

*Cho, choline-containing metabolites; Cr, creatine; Glx, glutamate/glutamine; 1H-MRS, proton magnetic resonance imaging; MADRS, Montgomery–Asberg Depression Scale; NAA, N-acetylaspartate*.

*Withdrawal was the first time point over when antidepressant medication was tapered with the intention to discontinue medication*.

Inspection of beta coefficients, risk ratios with their 95% confidence intervals (CIs), *t*-value approximations, and Wald statistics of nine variables entered into the CPH model indicated significant contributions of only two variables: changes in NAA/Cr and Cho/Cr. This was further confirmed when those nine variables were entered into the CPH forward stepwise likelihood ratio model, which consisted of two steps. After the inclusion of Cho/Cr change at the first step [B = –4.812; SE = 2.041; Wald = 5.559; d.f. = 1; *p* = 0.018; Exp(B) = 0.008], the final model (–2 log likelihood = 125.64; χ^2^ = 10.1; d.f. = 2; *p* = 0.006) consists of two variables: Cho/Cr change [B = –5.075; SE = 2.144; Wald = 5.604; d.f. = 1; *p* = 0.018; Exp(B) = 0.006] and NAA/Cr change [B = –1.870; SE = 0.935 Wald = 4.003; d.f. = 1; *p* = 0.045; Exp(B) = 0.154]. Parameter estimates of the final model (χ^2^ = 9.31296; d.f. = 2; *p* = 0.00951) are displayed in Table 4.

**Table 4:** Parameter estimates of the final model.

Factor	Relative risk (95% confidence interval)	*p*
**1H-MRS metabolite changes 6 months after recovery**		
NAA/Cr	0.1541 (0.0247–0.9628)	0.0455
Cho/Cr	0.0063 (0.0001–0.4178)	0.0179

**After dichotomization (only rise or fall monitored) 1H-MRS metabolite changes 6 months after recovery**		
Cho/Cr remains equal or rises	1.0000	
Cho/Cr decreases	2.7383 (1.1233–6.6752)	0.0267

*Cho, choline-containing metabolites; Cr, creatine; 1H-MRS, proton magnetic resonance imaging; NAA, N-acetylaspartate*.

We performed the next step to simplify the model by only analyzing the direction of neurometabolite changes between two periods. Dichotomization of variables and inclusion of composite risk of concomitant Cho/Cr and NAA/Cr directions of changes after remission did not significantly improve the model. The final dichotomized model consists of Cho/Cr decrease after remission as a highly predictive variable for depression recurrence. The results indicate that patients with decreased Cho/Cr after remission have a 2.7-fold higher risk of experiencing recurrence than patients in whom Cho/Cr ratio is stable or increased.

Escitalopram was the most frequently used antidepressant in both groups (five in the group of patients resolving with withdrawal, six in the recurrent episode group). Other antidepressants used in the group that started tapering off were fluoxetine (four patients), sertraline (4), venlafaxine (3), amitryptiline (2), reboxetine (1), citalopram (1), fluvoxamine (1), paroxetine (1), and mirtazapine (1). Other antidepressants used by patients who experienced a recurrent episode were sertraline (5), mirtazapine (2), fluoxetine (1), venlafaxine (1), reboxetine (1), citalopram (1), fluvoxamine (1), paroxetine (1), and imipramine (1).

Overall, the MADRS score significantly improved from the beginning of an episode to the start of the recovery phase, with no difference between patients who experienced a recurrent episode (20.3; SD = 4.1) and those who entered the withdrawal phase (20.4; SD = 3.8; *p* = 0.93). At the 6-month follow-up period, MADRS changes were only minor: (0.2; SD = 1.7) on average in subjects who entered a tapering-off period and (–0.3; SD = 1.6) in patients who experienced a recurrent episode later on, with no significant differences between those groups (*p* = 0.35).

## Discussion

To our knowledge, this is the first study to identify 1H-MRS correlates of risk of a subsequent depressive episode while on maintenance therapy in the recovery phase. We demonstrated that a decreased Cho/Cr ratio at the beginning of recovery is predictive of depression recurrence in patients on maintenance therapy. Decreased Cho/Cr was associated with a 2.7-fold risk (95% CI: 1.12, 6.68) for a recurrent episode. Our results also suggest that decreases in Cho/Cr and NAA/Cr ratios after recovery are related to a higher risk of the recurrent episode during maintenance therapy. Those risks persisted 6 months after index episode remission without a time-varying effect. Therefore, the observed findings could not be attributed to imminent worsening of patient’s condition that would closely follow decreases in Cho and NAA. In reviewing our results, we were faced with the difficulty of not being able to locate similar studies to directly compare our results.

NAA/Cr increases in frontal brain regions after SSRI therapy were previously reported (11, 13), which is in agreement with our findings. Most studies assessed patients until remission or soon after, but none followed up patients after recovery was achieved. Although the direction of NAA change is concordant with previous findings, the notable result is that NAA continued to rise in the favorable response group after recovery and remission while the patients continued pharmacotherapy. This could be of particular importance for future neuroplasticity studies.

Proton magnetic resonance spectroscopy research has provided relatively clear evidence about Cho increases in MDD patients (28, 29). We expected to observe a change in Cho; however, that hypothesis was based on consistent findings of increased Cho preceding the recovery phase. Intriguingly, the results indicate an increased membrane turnover rate in the DLPFC of non-recurrent patients. The observed ratio could presumably be consequent to PC-to-GPC mediated synthesis-to-breakdown overbalance, a finding congruent with the amygdala-to-DLPFC functional connectivity shift observed in neuroimaging studies.

When our finding of a continued Cho rise after the recovery phase was viewed together with NAA changes, there was ground for assuming a longer-term medication-induced neuroprotective effect. As symptom severity after recovery remained relatively stable, it is of particular importance that this postulated effect did not closely mirror clinical features. The combined increase of a marker of neuronal integrity (NAA) and a marker of membrane turnover (Cho) in non-recurrent patients points in the same direction as functional imaging studies showing that initial hypoactivity in the DLPFC of depressed patients turns to hyperactivity in response to treatment (28). It could also be speculated that neuronal degeneration, as demonstrated by neuronal loss in cross-sectional and longitudinal structural MR studies in depression, may be reversible, at least on a microstructural level.

Our results show that brain metabolic changes occur beyond the acute treatment and remission phases. In practical terms, that means metabolic changes continue for at least 6 months after substance exposure started. We do not have any clear assumption regarding how long those changes will continue, but this certainly warrants longer-term MRS studies.

Patients who converted to bipolar disorder were entered into the analysis, despite our awareness that they do not belong to the recurrent depression population. The rationale for this is that no patient in our analysis was followed up indefinitely, so a potential conversion to bipolar disorder could not be excluded for any single patient. We considered that this approach ensures sample homogeneity in the analysis, as the population of interest was patients with documented recurrent depression at the time of the index episode. By excluding bipolar patients, we would superimpose future knowledge to historical time points.

Due to a limited range of episode severity in our sample, we could not perform assay analysis to assess the degree to which observed metabolite changes are influenced by the baseline severity of an episode. We also did not include some other identified risk factors of recurrence, such as family history of recurrent depression, previous history of dysthymic disorder, and residual depressive symptoms prior to the current episode, as we consider that information is not fully reliable when sourced from a patient rather than medical documentation.

The retrospective design is a drawback of our study. Repeating the analysis prospectively could improve sample stratification and allow the inclusion of more detailed assessment instruments. Ideally, subject MRS evaluation would be performed at baseline, before therapy initiation, at remission, and throughout recovery. More scans would be helpful to eliminate the influence of temporal fluctuations, and would therefore improve the model’s sensitivity and specificity. With regards to the latter, although our sample size was relatively large for an MRS study, it was still underpowered to depict meaningful clinical sensitivity and specificity. Furthermore, the risk of type II error could not be eliminated, so repeated studies with similar design are certainly needed.

An important limitation of our study is that patients received different pharmacological treatments. It was only designed to correlate neurochemicals with clinical features, and no direct inference could be made to a specific antidepressant class. Current knowledge on subclass and substance-specific effects on clinical features is limited, and the evidence is even weaker for *in vivo* neurochemical levels. However, such effects exist, as clearly demonstrated in a report of the opposite effects of escitalopram and reboxetine in healthy subjects (30).

The formal definition of recovery is rather imprecise, which limits the possibility of generalizing our results to a specific temporal pattern. Still, our primary aim was to investigate whether brain metabolite changes near the remission period delineate the future course of the depressive episode. This is obviously the case, but there is a need to clarify whether Cho and NAA changes are directly related to pathophysiological processes determining the course of the disorder or if they are only indirect correlates.

In the longer term, identifying the prognostic correlates of the disorder’s course is important to delineate the neurophysiological background of depressive disorder and possibly improve patient wellbeing by reducing the risk of recurrent episodes.

Research of 1H-MRS metabolite changes over a longer recovery phase is required to provide more detailed insight into the relationships of measurable 1H-MRS metabolites as putative markers of the longitudinal course of the depressive disorder. Our findings support the notion of other researchers (28) that targeting neural plasticity in depression may lead to treatment breakthroughs.

## Ethics Statement

This study was carried out in accordance with the recommendations of Guidelines for neuroimaging research, Committee for Medicines of the Polyclinic Neuron with written informed consent from all subjects. All subjects gave written informed consent in accordance with the Declaration of Helsinki. The protocol was approved by Ethics Committee of the Polyclinic Neuron.

## Author Contributions

NH substantially contributed to conception and design, to the acquisition of data, to analysis and interpretation of data, drafting the article, critically revising the article for important intellectual content, and provided final approval of the version to be published. HŠ, DO, TF, and VET substantially contributed to acquisition of data, and critically revised the article for important intellectual content. MAR and MIR substantially contributed to conception and design, to the acquisition of data, to analysis and interpretation of data, and critically revised the article for important intellectual content. PH substantially contributed to conception and design, to analysis and interpretation of data, drafting the article, and critically revising the article for important intellectual content. MBJ and BR substantially contributed to acquisition of data, and critically revised the article for important intellectual content. PK substantially contributed to conception and design, to the acquisition of data, to analysis and interpretation of data, drafting the article, and critically revising the article for important intellectual content.

## Conflict of Interest Statement

NH, PK, and MBJ have participated in clinical trials sponsored by Otsuka, Eli Lilly, Forest Laboratories, Lundbeck, Takeda, Allergan, GlaxoSmithKline, and Pfizer. The reviewer NY and handling editor declared their shared affiliation.
